# Decreasing Quality of the New Generations of Anti-Müllerian Hormone Assays

**DOI:** 10.1155/2014/165352

**Published:** 2014-03-11

**Authors:** Krzysztof Lukaszuk, Beata Ludwikowska, Joanna Liss, Michal Kunicki, Miroslaw Sawczak, Aron Lukaszuk, Lukasz Plociennik, Grzegorz Jakiel, Tomasz Wasniewski, Izabela Woclawek-Potocka, Dorota Bialobrzeska

**Affiliations:** ^1^INVICTA Fertility and Reproductive Centre, 80-850 Gdansk, Poland; ^2^Department of Nursing, Medical University, 80-952 Gdansk, Poland; ^3^Department of Obstetrics and Gynecology, Faculty of Medical Sciences, Varmia and Masuria University, 10-561 Olsztyn, Poland; ^4^INVICTA Fertility and Reproductive Centre, 00-019 Warsaw, Poland; ^5^Department of Photophysics, IFFM Polish Academy of Sciences, 80-952 Gdansk, Poland; ^6^Department of Obstetrics and Gynecology, The Medical Center of Postgraduate Education, 00-416 Warsaw, Poland; ^7^Department of Reproductive Immunology and Pathology, Institute of Animal Reproduction and Food Research, Polish Academy of Sciences, 10-747 Olsztyn, Poland

## Abstract

Anti-Müllerian hormone (AMH) measurements are widely used to optimize the stimulation protocols. First generation AMH kits correlated well with ovarian reserve and response to stimulation. In the present study we aimed to asses if the new generation kits share the same accurate correlations. Retrospective data were collected from 8323 blood samples. For comparison we used Immunotech I generation kit (ImI 4035 samples), Beckman Coulter II generation kit RUO (BCII RUO 3449, samples) and Beckman Coulter II generation kit with IVD certificate (BCII IVD 839 samples). We compared average AMH concentrations measured with different kits, as well as correlation between kits. We also compared average AMH concentrations in sera collected on different cycle days and samples of different quality of preservation. AMH serum concentrations differed for each kit, ranging 4.4 ± 4.12 (mean ± SD) for the ImI, 2.68 ± 3.15 for the BCII RUO, and 1.64 ± 2.85 for BCII IVD. The mean differences from an adjusted regression model were −48.7%, −40%, and −69.2%, respectively. In conclusion, the changes of the BC AMH kits are unpredictable; however, the improvement of them is still possible. It would be very dangerous to use elaborated stimulation protocol (based on the Ist generation AMH results) with the results from the IInd generation assays.

## 1. Introduction

Anti-Müllerian hormone (AMH) is a member of the transforming growth factor-*β* superfamily. In nature it exists as a glycoprotein dimer and this chemical structure affects its function and detection method [1, 2]. It is primarily produced by granulosa cells in primordial, preantral, and antral follicles [3, 4]. Although serum level of AMH is about 10–100 times lower than in the place of its production (antral follicle fluid) and far from the level of potential biological activity, it is well correlated with the pool of antral follicles and this correlation implicates its clinical usefulness [5, 6]. That is why it is well established as the best ovarian reserve marker [7–12]. There is good correlation between the amount of antral follicles and the response of patients to stimulation protocols [13–16]. However, the correlation between AMH and IVF main outcome (live births) has still not been confirmed [15, 17–19].

It is well known that AMH measurements were very useful for different reasons and for different investigated populations. As a predictor of diminished ovarian reserve it was less useful in young patients as screening test but it was a powerful tool for middle aged and older patients (higher risk populations) as test to establish diagnosis because of high levels of both sensitivity and specificity. But mostly, AMH measurements were widely used to optimize the stimulation protocols. It was well correlated with ovarian response to stimulation. There were few publications emphasizing the decision-making process based on AMH serum concentrations [20].

The importance of the AMH measurements caused the need for commercialization of the kits. Beckman Coulter Company consolidated the available systems from Immunotech Ltd. and Diagnostic Systems Ltd (DSL). They were based on different monoclonal antibodies and also different standards used. They were integrated into the Beckman Coulter II generation kit. According to information from the manufacturer, the AMH Gen II ELISA kit uses the same antibody as in the DSL kit, but with the standards of the Immunotech assay kit [21]. Unfortunately, the results began to correlate poorly in clinical situations. These signals were reported by different clinicians (but also patients) and caused doubts among them. Taking above concerns into consideration, we decided to revise our quality management data. Therefore, this paper represents a retrospective study of our results.

## 2. Materials and Methods

A total of 8323 blood samples from different women were taken into consideration. Samples were obtained from women of 12–62 years of age being seen for investigation of infertility or fertility preservation reasons requiring AMH assessment. Most of the samples were collected at the Fertility Clinic Invicta—6259 patients in Warsaw and Gdansk from the beginning of 2007 till December 2012. Additionally, a total of 2064 samples were collected from different clinics during this period. The tests were done sequentially and each patient was tested only once using one kit. The AMH concentration in the blood plasma was measured from 2007 till April 2011 with an Immunotech I generation kit (4035 samples) and from August 2010 till August 2012 with a Beckman Coulter II generation kit RUO (3449 samples). From August 2010 till April 2011, both tests (Immunotech I generation and Beckman Coulter II generation RUO) were used in parallel depending on the availability from the supplier. From August 2012 till December 2012, we were supplied with the same Beckman Coulter II generation kit but with an IVD certificate (839 samples).

Blood samples were taken between first and fifth day of the menstrual cycle in 47% of cases, when patients attended the clinic for the routine first visit. They were collected aseptically into tubes with clotting activator, vacuum blood collection system Vacutainer Becton Dickinson. The blood collection was on different days of the cycle in 28% of the samples. The results from 25% of the samples from different fertility clinics were sent to us without the information about the women's cycle days. The serum for AMH assay was separated within 2 hours from venipuncture and frozen in aliquots at −80°C until it could be analyzed in batches. Samples that were lipaemic or haemolysed and samples not frozen within 2 hours of venipuncture were excluded from the study.

AMH was measured using ELISA kits according to the manufacturer's instructions. EIA AMH/MIS (catalogue number A16507) (Immunotech, Marseille, France) has sensitivity of 1 pmol/L and reported intra- and interassay coefficients of variation of less than 12.3% and 14.2%, respectively, according to the product insert. The Beckman Coulter Gen II RUO assay (catalogue number A73818) (Beckman Coulter Inc. Brea, CZ 92821 USA) and The Beckman Coulter Gen II IVD assay (catalogue number A79765) (Beckman Coulter Inc. Brea, CZ 92821 USA) both have sensitivity of 0.57 pmol/L and reported intra- and interassay coefficients of variation of less than 5.4% and 5.6%, respectively, according to the products' inserts. We were informed by the manufacturer that there was no difference between the BCII RUO and BCII IVD kits, except for the label.

## 3. Sample Processing

Collection and handling of all AMH samples were conducted according to the standards set by the manufacturers and did not vary between different assays. Serums samples were transported immediately to the Invicta Routine Laboratory and separated within 2 hours. Samples were frozen in aliquots at −80°C until analysis, normally within 3-4 days of receipt. The laboratory participates in the External Quality Assurance Schemes for Reproductive Medicine (from 2010 till 2012), which confirms its satisfactory performance.

Clinical data were collected retrospectively using an electronic database (Invictus ver. 3.3.3, Invicta Ltd., Poland). We took into consideration age, menstrual cycles (duration, regularity, and bleeding), the interval between blood collection for AMH, and the beginning of stimulation.

We acquired laboratory data from laboratory software (Invictus Laboratory ver. 2.1.3, Invicta Ltd., Poland). We obtained the exact day of the cycle when blood was collected, the time of collection, and the history of sample trip from the blood collection point to the result (the duration and temperature of the sample transportation, time and temperature of the centrifugation, the period between serum collection and freezing, the duration of freezing, and time from thawing till getting results).

The study was approved by the Local Research Ethics Committee (the Varmia and Masuria Chamber of Physicians).

## 4. Statistical Analysis

Data analysis was performed using StatSoft, Inc. (2011) STATISTICA (data analysis software system), Version 10. http://www.statsoft.com/.

Quality control was shown as mean concentration against the expected one with standard deviation, coefficient of variation, and biases.

The characteristics of the investigated assay groups were compared using Mann-Whitney* U* test.

The age-related relationship of the three assays to AMH was visualized using scatter plots and quadratic fit on a logarithmic scale. The age-adjusted regression analysis was used to estimate the difference in AMH serum concentrations between investigated assays. Influence of the blood collection cycle day on AMH serum concentration was analyzed using Mann-Whitney* U* test.* P* value of <0.05 was considered statistically significant.

## 5. Results

The analytical characteristics of AMH tests according to manufacturer show the linearity of the ImI test from 0.1–21.0 ng/mL for ImI to 0.08–22.5 ng/mL for both of BC II tests. They are comparable but 6.8% lower in range for Immunotech 1st.

Table 1 compares the quality controls of all tests performed in our laboratory. We did not find any problems using tests with the manufacturers' controls. All of them were marked as good by external quality control company.

### 5.1. The Characteristics of the Results Received on Three Different Kits

**Figure 1:**
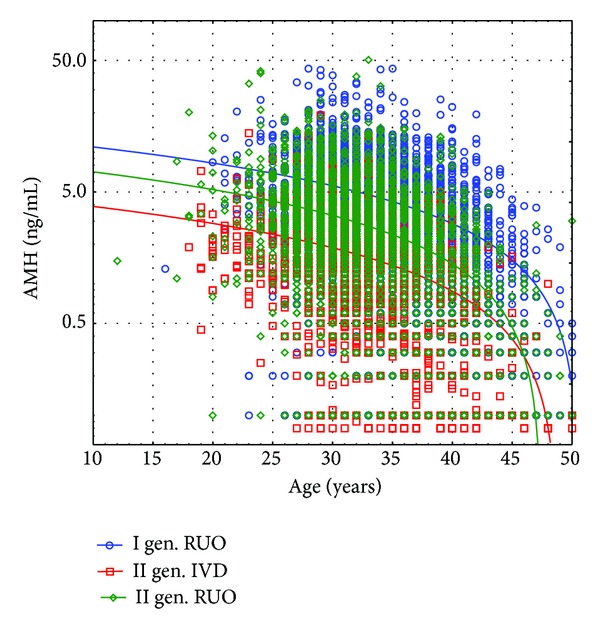
Unselected AMH values from Immunotech I (blue line), Beckman Coulter gen. II RUO (green line), and Beckman Coulter II IVD (red line) assays as a function of age. Lines show the regression fits of AMH serum concentration against a quadratic function of age.

There are statistically significant differences between each group in AMH serum concentrations, as well as age (Table 2). The average ages of the groups differ significantly. The groups measured by II generation assays were younger and should be effective in higher AMH results but AMH results were much lower comparing to those received with the I generation kit. The difference between both II generation assays results was also very big which is not possible to be explained by the patients' small age difference only.  Figure 1 shows the correlation of AMH with age for the unselected groups.

**Figure 2:**
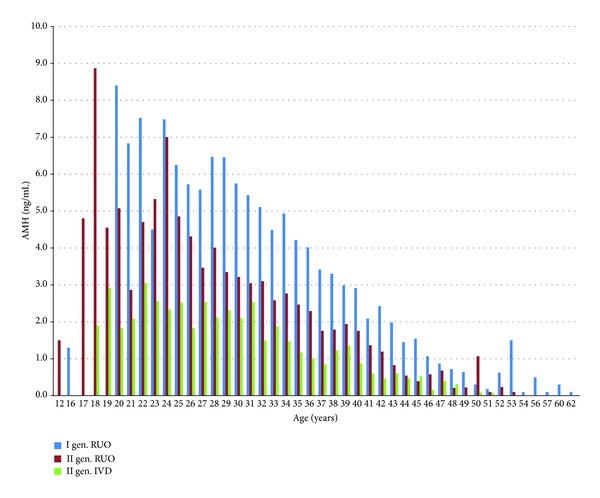
Mean values of AMH of three different tests as a function of age. We can see the decrease in the mean values of each consecutive test placed on the market.

For the estimation of the difference between the expected results among the assays, we adjusted the ages of the investigated groups (Table 3). We found an approximately 50% difference between 1st and 2nd generation assays and an approximately 70% difference between the 1st generation and the last version of the 2nd generation Beckman Coulter kits. The difference between both 2nd generation kits was 40%, which contradicts the manufacturer's information about the identity of both assays. These differences are visible in each investigated age group as shown in Figure 2. The amounts of the analysed samples performed in each women ages were proportional for all analyzed kits—data not shown.

Figure 1 demonstrates unselected AMH values from Immunotech I, Beckman Coulter gen. II RUO, and Beckman Coulter II IVD assays as a function of age. Lines show the regression fits of AMH.

Figure 2 presents mean values of AMH of three different tests as a function of age. We can see the decrease in the mean values of each consecutive test placed on the market.

We found no differences in average AMH results in days 1–5 and day 6 through the end of the cycle (Table 4). We found lower results from the 1st generation tests' results from the other clinics where the quality of the samples protection and transportation were unknown.

## 6. Discussion

The aim of our work was to compare different AMH measurement kits.

AMH is a very useful marker for estimating the ovarian reserve in women. As we demonstrated before, the results obtained using I generation kits correlated well with the stimulation effects (own data submitted to Reproductive Biology). The clinical decisions of stimulation protocols were simple and safe. In the previous study we found positive correlation between AMH serum concentrations and life-birth rates assessed in multivariate regression analysis [22]. Moreover, other authors also reported good correlation between single AMH measurements and stimulation results using generation I assays [20].

There are lots of proposals of AMH serum concentrations use in clinical practice. Up to now, our clinical decisions were routinely based on AMH serum concentrations. However, we noticed that the correlation between the AMH serum concentration measurements and clinical results in our clinic decreased in subsequent years. Moreover, we received signals of problems from our laboratory, where AMH is the accredited measurement with all necessary additional quality controls.

That is why we decided to compare our results from different generations of kits. We started to look more carefully at the correlation between AMH serum concentrations and clinical practice. The introduction of the second generation of AMH kits changed our clinical practice. We were informed by the manufacturer about the changes in results that we should expect. The AMH results should have been lower by approximately 30% on average. The compared groups were of different ages, but the group with BCII RUO was younger than the first group, which should have even increased received results comparing to ImI (Table 3). The comparison was made after adjustment of the age. The generation II RUO AMH results were totally different than before and had lost their correlation with clinical situation (unpublished data). We found the results of AMH 48.7% lower on average (Table 4). The next kit, which was introduced by Beckman Coulter in August 2012, was introduced as being the same with the changes of the label only. It ceased being labelled RUO and was dedicated for clinical use (IVD). The results were diminished once again. We found another 40.0% decrease of the mean results that gave us a 69.2% decrease compared to the average results of the 1st generation kits. We have to remember that we were informed by Beckman Coulter about constant results from the both generation II kits.

Rustamov et al. [23] suggested that the decrease in AMH serum concentrations obtained using Gen II assays could have been caused by degradation of the specimens in one or both assays. We could not have confirmed this assertion because we had been using the same procedure, the same equipment, and the same staff. All samples in this retrospective study were subjected to the same handling procedures and analysed by the same laboratory. We, as the clinicians, use AMH measurements as a routine first step in the infertility investigation procedure. No other alterations in our practice have happened contemporaneous with assay change. We are working on one system where each bias in the stimulation protocols and the results are directly reported to board and corrected immediately. We reported our problems to Beckman Coulter, but we did not receive any reliable information.

Some authors have reported the variability of the results between assays [23]. They concluded that they were the results of marked degree of sample instability seen in the laboratory. They excluded biological variation, which was confirmed to be small [24, 25], and the intra-and interassay variation (which they found <5%) [23].

We found good intra- and interassay variations in all kind of kits. We conclude that most of biases that we found were probably connected with the manufacturer's technological problems.

We also wanted to find out whether the AMH results are dependent on the cycle day when the blood is collected and on the quality of the samples protection and transportation. We did not find the differences between the average results from different cycle days of blood collection from our lab independently from the assays type. For the generation II kits, we also did not find the difference between the results of our optimized laboratory procedure and the results of the unattended samples of unknown quality. The results from the 1st generation kit were lower in the unattended samples, even after age adjustment. This can be explained by degradation of the sample that could have happened in the higher rate during the unattended transportation. On the other hand, Rustamov et al. found the increase in the AMH concentration after the room temperature storage [23]. Those authors performed all analyses using the Gen II systems. The increase in the AMH concentration can be explained by the second binding site on the antigen being exposed by the dissociation which could be undetected by the II generation kits. This is the first study to report the decrease in sensitivity and the diminished average level of AMH when using II generation kits. We found our results to be very important and urgent for clinicians. It would be very dangerous to use elaborated stimulation protocol relaying on the results from the II generation kits. Moreover, we presume that relaying on those results would lead to the increase of patients suffering from OHSS as the reason for the application of the inappropriate high doses of gonadotropins for stimulation. Finally, relaying on the lower levels of AMH while making decisions about the stimulation method can lead to higher percentage of patients suffering from OHSS.

## Figures and Tables

**Table 1:** Quality control of each analyzed AMH test.

Control	Expected conc. (ng/mL)	Imprecision between run					Trueness	
		Mean Conc. (ng/mL)	Min	Max	SD	CV%	Bias	Bias%
AMH/MIS ELISA Immunotech REF A16507								
Controls 1	0.42	0.4	0.24	0.49	0.06	13.4	−0.05	−0.3
Controls 2	11.3	11.4	10.5	12.3	0.48	4.2	0.01	0.6

AMH Gen II ELISA Beckman Coulter REF A73818								
Controls 1	3.0	3.09	2.4	3.6	0.26	8.3	0.03	3.0
Controls 2	9.0	9.05	7.6	10.6	0.75	8.3	0.01	0.5

AMH Gen II ELISA Beckman Coulter REF A79765								
Controls 1	2.9	2.86	2.51	3.11	0.22	7.7	−0.01	−1.4
Controls 2	8.2	8.47	7.44	9.65	0.58	6.8	0.03	3.3

CV: coefficient of variation, AMH: anti-Müllerian Hormone, and SD: standard deviation.

**Table 2:** The characteristics of the investigated groups and AMH results in each assay.

	AMH (ng/mL)			Age (year)		
	Imm. gen. I	BC gen II RUO	BC gen II IVD	Imm. gen. I	BC gen II RUO	BC gen II IVD
*N *	4035	3449	839	4035	3449	839
Mean	4.4	2.68	1.65	34.33	32.29	33.32
Std. dev.	4.12	3.15	2.85	4.95	5.09	5.63
Median	3.4	1.8	1.2	34	33	32
Upper quartile	5.9	3.8	2.1	37	36	36
Lower quartile	1.7	1.0	0.52	31	30	29

**Table 3:** Mean difference from an age-adjusted regression model expressed as a percentage difference (%).

Immunotech gen. I	−48.7 (−52.4 to −45.5)	−69.2 (−76.2 to −68.2)
BC gen. II RUO		
	−40.0 (−50.0 to −41.7)	
BC gen. II IVD		

**Table 4:** The mean (±standard deviation) of the different kits depending from the day of the cycle, from day 1 to day 5 of the cycle, from day 6 to the end of the cycle, and all known cycle days together and results from unknown cycle day.

	Cycle day	*N *	Mean ± SD	Median	95% CI	*P *
Immunotech I gen RUO	1–5	1845	4.56 ± 3.86	3.7	2.0–6.2	0.07
	>5	856	4.51 ± 4.51	3.3	1.6–5.9	
	Known	2701	4.55 ± 4.08	3.6	1.9–6.1	<0.001
	Unknown	1334	4.11 ± 4.21	3.0	1.3–5.5	

BC II gen RUO	1–5	1446	2.6 ± 2.91	1.8	0.9–3.3	0.69
	>5	1097	2.67 ± 2.9	1.9	0.8–3.4	
	Known	2543	2.63 ± 2.91	1.8	0.8–3.4	0.91
	Unknown	906	2.84 ± 3.74	1.9	0.8–3.7	

BC II gen IVD	1–5	358	1.62 ± 1.65	1.3	0.6–2.0	0.55
	>5	287	1.62 ± 1.69	1.1	0.5–2.2	
	Known	645	1.62 ± 1.67	1.2	0.5–2.0	0.71
	Unknown	194	1.76 ± 2.07	1.2	0.5–2.2	
